# Mechanical Properties of Concrete Containing Liquefied Red Mud Subjected to Uniaxial Compression Loads

**DOI:** 10.3390/ma13040854

**Published:** 2020-02-13

**Authors:** Gyeongcheol Choe, Sukpyo Kang, Hyeju Kang

**Affiliations:** 1 Department of Architectural Engineering, Chungnam National University, 99 Daehak-ro, Yuseong-gu, Daejeon 34134, Korea; choegc@cnu.ac.kr; 2 Department of Architecture, Woosuk University, Jincheon, 27841, Korea; leekang02@nate.com

**Keywords:** cement type, liquefied red mud, mechanical property, stress–strain curve, compression load

## Abstract

This study used liquefied red mud (RM) sludge, an aluminum industry by-product, as a construction material. Accordingly, various methods were examined that used the fabricated liquefied red mud (LRM) as an admixture for concrete, and the mechanical properties of concrete were then evaluated according to the cement type and the amount of LRM. The LRM mixing methods (replacement and addition) were compared, and the slump and compressive strengths of concrete were evaluated for each method. To examine the mechanical properties according to the cement type and the amount of LRM, two types of cement (ordinary Portland cement and slag cement (SC)) were used, and 20 and 40 wt% LRM (with respect to the cement weight) were added. The mechanical properties of the stress–strain curve (SSC), compressive strength, peak strain, and elastic modulus were quantified. When the slump and compressive strength of concrete were considered based on the experimental results, the addition LRM mixing method was recommended as the appropriate method for LRM. As the addition of LRM increased, the mechanical properties of concrete degraded. However, when SC was used, the mechanical properties did not significantly change when different amounts of LRM were added (up to 20%). In addition, the SSC of LRM concrete could be approximated based on the use of the relationship of the compressive strength and peak strain according to the cement type and the amount of LRM.

## 1. Introduction

Red mud (RM) is an aluminum industry by-product that is generated during the process of extraction of alumina from bauxite ore. It is discharged in a high-alkaline state at a pH in the range of 10–13 owing to the influence of caustic soda (NaOH) which is added in the process [1,2]. In general, 1–1.5 tons of RM are generated when a ton of alumina is produced. Accordingly, 90–150 million tons are estimated to be discharged each year worldwide [3,4]. To this date, large amounts of RM have been managed, either by (a) storing them, (b) using them as landfill in open fields, or by (c) dumping them at sea. It has been reported that various heavy metals contained in RM and their high pH values may cause various environmental problems [3,5].

The continuous industrial growth depends on the stable treatment of waste and the conservation of natural resources [5]. Therefore, in the global aluminum industry, the treatment and effective recycling of RM—which has minor influences on the natural environment—has attracted increased attention [6]. Fortunately, industrial by-products can be recycled as raw materials in replacement of natural resources because many of them contain minerals that can be obtained from natural resources. RM, which contains alumina, iron oxide, and titanium, can also be recycled in various forms in different industries as other industrial by-products. In fact, the methods of recovering and reusing rare earth elements contained in RM, or the use of RM itself as a pigment and an additive for wastewater treatment and soil remediation, have also been introduced [4].

In the construction industry, the reduction of CO_2_ emissions from cement production has been a major issue. Cement is one of the most important materials in the construction industry, and has been extensively used in many countries. The production of one ton of clinker is estimated to emit one ton of CO_2_ gas. Additionally, it has been reported that approximately 7% of global CO_2_ emissions is related to cement production [7]. In the construction industry, the most effective method used to reduce CO_2_ emissions is to decrease the use of cement. Industrial by-products, such as fly ash, ground granulated blast-furnace slag, and silica fume, have been used as admixtures for concrete. RM is an industrial by-product, and can be used as an admixture for cement-based materials, such as mortar and concrete [8,9]. Moreover, if the RM is used as a construction material, it can be utilized in large quantities, and the environmental problems caused by its disposal can be solved [9,10,11,12,13].

In many prior studies, the effects of RM on the fluidity, hardening properties (microstructure, water absorption, and mechanical properties), and durability (chloride penetration and whitening) of mortar and concrete have been experimentally evaluated [10,14,15,16,17,18]. A number of studies, however, reported experimental results in which the mortar or concrete that used RM exhibited degraded fluidity and mechanical performance [10,15]. The physical properties of RM, whose particles are smaller than those of cement, have been pointed out as the cause. As RM adsorbs more moisture than cement in the unhardened state, the fluidity of concrete is degraded [17]. In addition, cement-based hardening bodies mixed with RM have high-water absorption and low-mechanical performances because they contain many capillary pores with diameters in the range of 10 to 1,000 nm [15,19]. Despite these problems, it has been confirmed that mortar or concrete mixed with RM can attain the required mechanical performance for a construction material if the application conditions of RM are properly selected. Thus, RM is still an industrial by-product with increased potential for use as a construction material. To accomplish the active use of RM in the construction industry, it is imperative that it is properly used, and that various examinations are conducted in terms of material composition, including the cement type.

RM is discharged as sludge from the Bayer process with a moisture content of 50%. It is very difficult to manage constant moisture content because it is significantly affected by environmental conditions [19]. Therefore, RM, which is used as an admixture for mortar and concrete, is utilized as dried powder after high-temperature drying and grinding processes. These processes are energy-intensive steps that require additional energy input. They cause increases in the manufacturing costs and hinder the recycling of RM [20]. 

Therefore, in this study, we fabricated liquefied red mud (LRM) to achieve the efficient quality control of RM and the omission of the energy-intensive drying process. In addition, the proper use of LRM as concrete admixture was examined to expand the use of RM as a building material. The mechanical properties of concrete mixed with LRM are presented based on the evaluation of the compressive strength, peak strain, elastic modulus, and stress–strain relationship of concrete according to the cement type and the amount of LRM.

## 2. Materials and Methods

### 2.1. Materials

#### 2.1.1. Liquefied Red Mud

**Figure 1:**
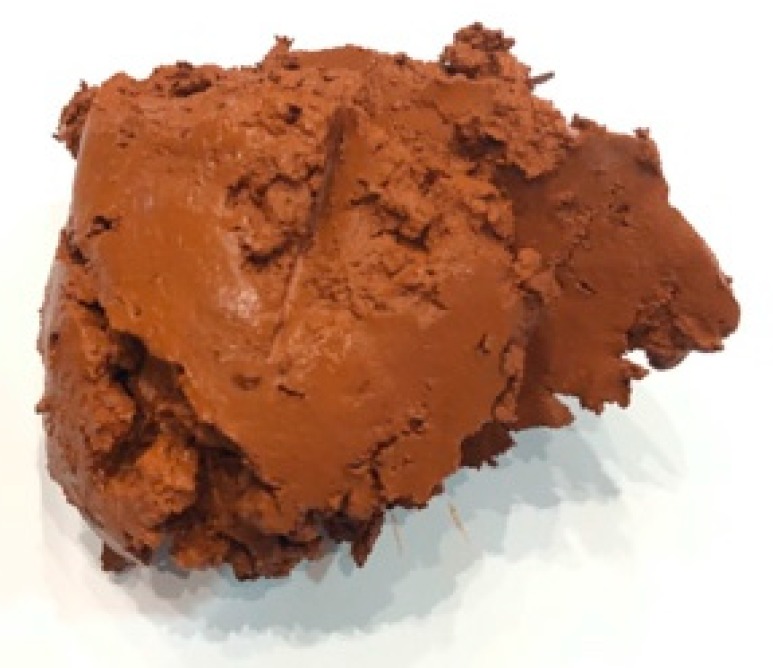
Red mud sludge.

LRM was fabricated with the use of water, RM sludge, a thickener, and an antifoamer. The RM sludge (Figure 1) obtained from an alumina factory located in Yeongam-gun, Jeollanamdo, South Korea was used. Table 1 shows the physical properties and chemical composition of the RM sludge. The RM sludge had a density of 2.0 g/cm^3^, an average diameter of 4.31 μm, a moisture content of 50.2%, and a viscosity of 20,000 cPs. The main oxides contained in RM were SiO_2_, Al_2_O_3_, Fe_2_O_3_, CaO, MgO, Na_2_O, and K_2_O, while the content of SiO_2_, Al_2_O_3_, and Fe_2_O_3_, represented more than 80% of the total mass. Table 2 shows the physical properties of the thickener and antifoamer. The thickener was a methyl cellulose type and the antifoamer was a polyoxyalkylene alkyl ether type, which was a transparent liquid.

**Figure 2:**
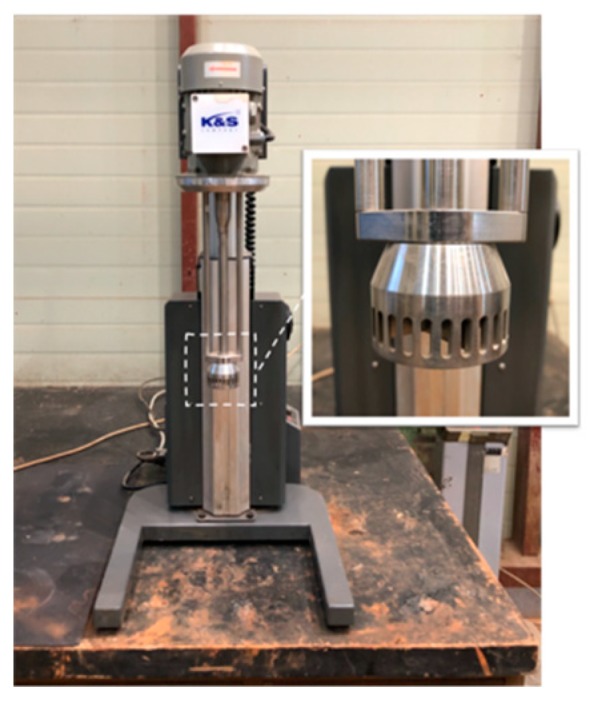
Homo mixer.

**Figure 3:**
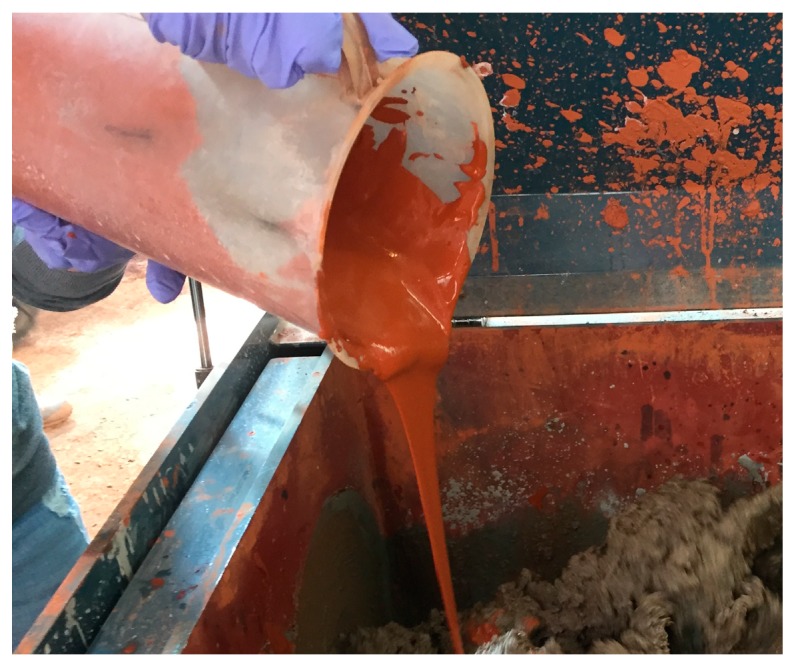
Liquefied red mud (LRM).

Table 3 shows the mix proportions of each material used to fabricate the LRM. The RM sludge, water, thickener, and antifoamer were mixed at a ratio of 1:0.5:0.0036:0.0014 based on the mass of the RM sludge. A homo mixer (Figure 2) was used for mixing. For the homo mixer, the shear energy formed in the fine gap between the rotor and stator creates a vacuum environment. When the RM sludge is sucked into the gap, the particles are dispersed, thereby reducing the particle size. After water and RM sludge were mixed for three minutes, the thickener and antifoamer were added and mixed for two minutes. Regarding the physical properties of the fabricated LRM (Figure 3), the solid content was 51.6% and the unit weight was 1570 kg/cm^3^. The average particle diameter was 3.15 μm and the viscosity was 7850 cPs. 

#### 2.1.2. Cement

Ordinary Portland cement (OPC) and slag cement (SC) were used. Table 4 shows the physical properties and chemical compositions of OPC and SC which were used to fabricate concrete.

#### 2.1.3. Aggregate

Table 5 shows the physical properties of the coarse and fine aggregates used to fabricate concrete. Crushed sand with a density of 2.56 g/cm^3^, maximum size of 5 mm, water absorption of 1.01%, and a fineness modulus of 2.6, were used as fine aggregates. Granite with a density of 2.67 g/cm^3^, a maximum size of 20 mm and water absorption of 1.39% were used as the coarse aggregate.

### 2.2. Experimental Plan and Concrete Mix Proportions

Table 6 shows the experimental plan and concrete mix proportions. Sixteen specimens were prepared for the experiment according to the cement type, water-binder (W/B) ratio, LRM mixing method, and LRM content. The method that replaced some of the cement with LRM, and the addition method that added LRM were first planned. The amounts of LRM substituted or added were 20% and 40% of the cement weight. The W/B ratio of concrete was set to 45% and 65%, each of design strength (f_ck_) was set as 35 and 15 MPa and SC was used. The slump was measured as a fresh property and the compressive strength as a hardened property.

For the evaluation of the mechanical properties of LRM-added concrete as a function of the cement type, two types of cement, i.e., OPC and SC were used. The W/B ratio was set to 55% (f_ck_ 25 MPa) regardless of the cement type. Regarding the LRM, 20% and 40% of the cement weight were added in the same manner as in the previous step. Regarding the mechanical properties, the stress–strain curve (SSC), compressive strength, elastic modulus, and peak strain, were evaluated. 

The sand-aggregate (S/A) ratio was set at 50% for all the concrete specimens used in the experiment.

### 2.3. Specimens

**Figure 4:**
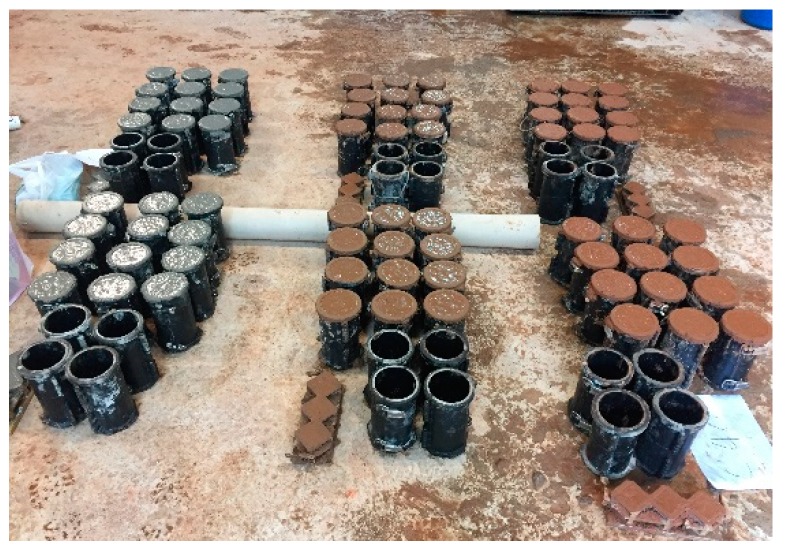
Fabrication of concrete specimens.

Figure 4 shows the fabrication of the concrete specimens. The specimens were fabricated in the cylindrical form with a diameter of 100 mm and a height of 200 mm in accordance with KS F 2403, “a method of concrete specimen fabrication for strength tests” [21]. The specimens were cured in the mold for 24 h and then in a water tank at 20 ± 2 °C until the test date.

### 2.4. Test Setup and Test Method

**Figure 5:**
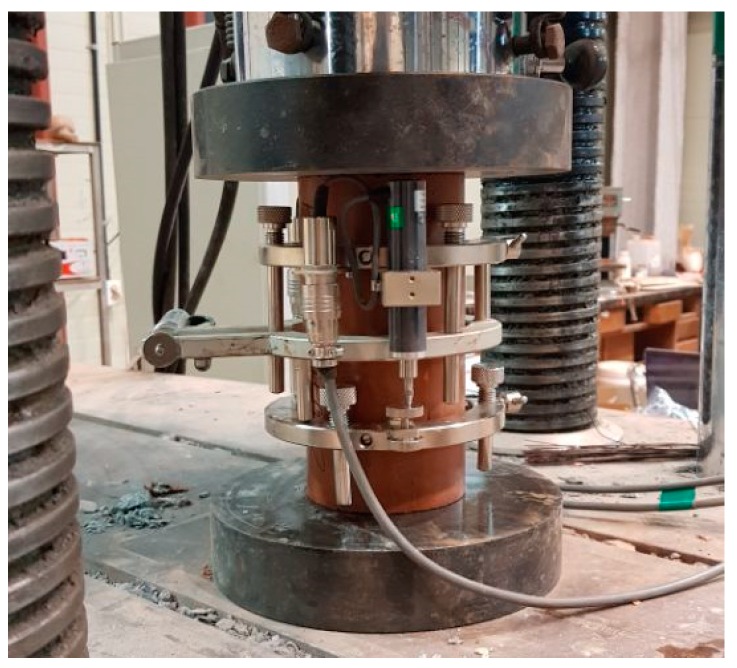
Test setup for the compression loading test.

**Figure 6:**
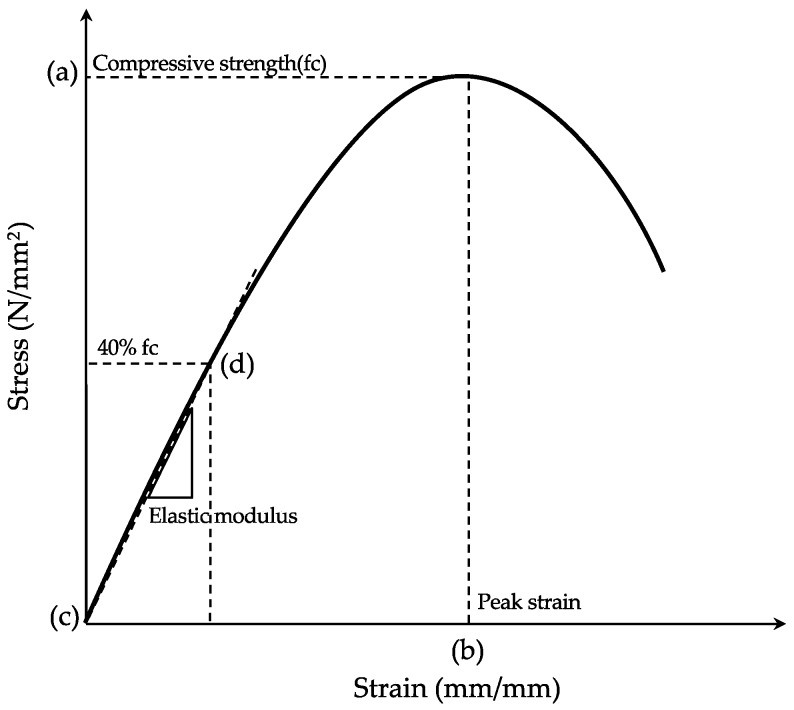
Definition of compressive strength, peak strain, and elastic modulus on stress–strain curve (SSC).

Figure 5 shows the test setup for the compression test. A universal testing machine (UTM) with a maximum capacity of 2000 kN was used. The compression load was applied at a rate of 0.3 N/mm^2^ per second, and the stress of each specimen was measured with a load cell. The deformation of each specimen under the compression load was evaluated using the average value of the displacement meters installed on both sides of the specimen. In the SSC obtained from the compression test (Figure 6), the compressive strength (a) was defined as the maximum stress, and the maximum strain (b) was defined as the deformation at the maximum stress. The elastic modulus, i.e., the sequence modulus, was estimated as the slope of the straight line which connected the initial point (c) and the stress point which corresponded to 40% of the maximum stress (d). Before the loading test, preloading was performed (in which 30% of the estimated maximum stress was applied three times) to reduce the influence of creep deformation on each specimen. In addition, the average values obtained from the three specimens were used as the outcomes of each test.

## 3. Experimental Results and Discussions

### 3.1. Properties of Fresh and Hardened Concrete According to the LRM Insertion Method

**Figure 7:**
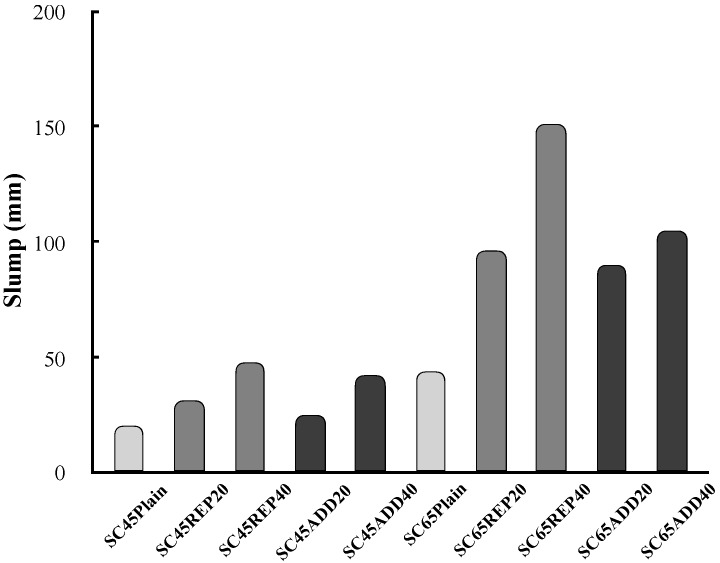
Slump of fresh concrete according to LRM mixing method

Figure 7 shows the slump of concrete according to the LRM mixing method. LRM-substituted concrete tended to show a larger slump than LRM-added concrete. It is generally known that the fluidity of mortar or concrete containing RM is significantly degraded. In this study, however, the slump increased as the amount of LRM increased, regardless of the mixing method. This difference can be attributed to the forms of RM used in each study. In most of the previous studies, dried RM powder was used. According to Tang et al. [17], RM—whose particles are smaller than those of cement—degrades the fluidity of concrete because it adsorbs more moisture in the fresh concrete state. For the LRM used in this study, however, sufficient moisture could be supplied in the fresh concrete state because approximately 48% of its total mass was composed of water. 

**Figure 8:**
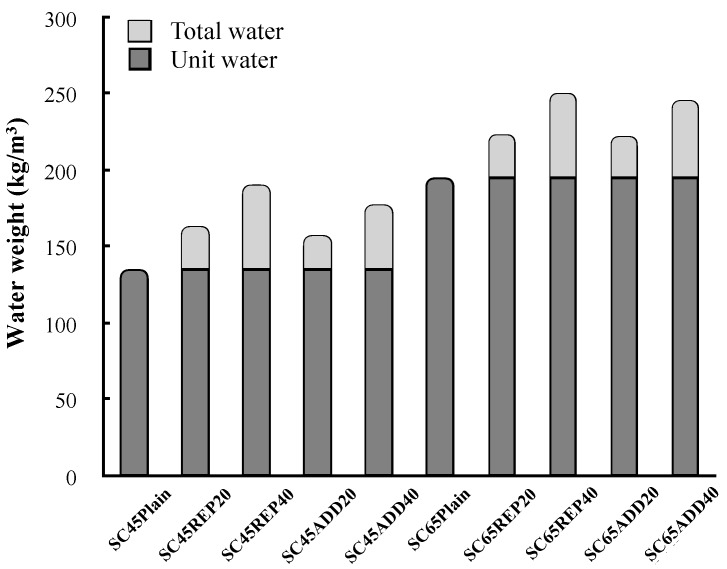
Water weight in mix proportion according to LRM mixing method

**Figure 9:**
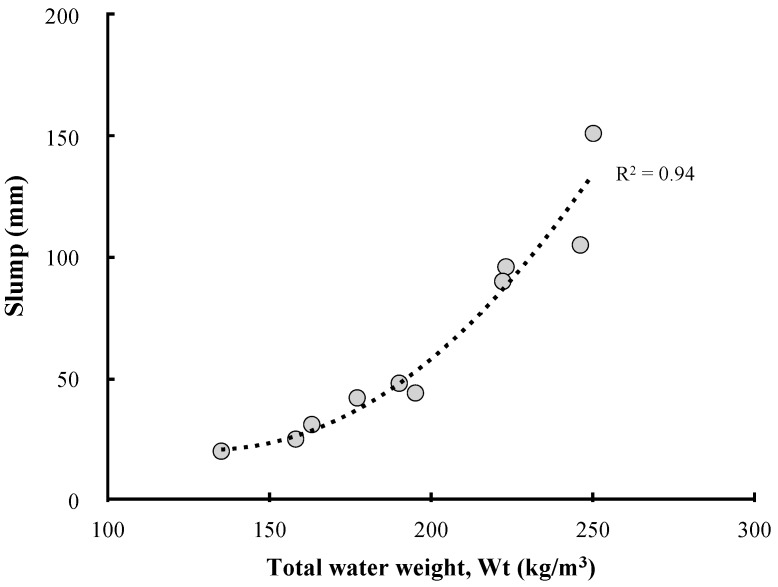
Relationship between slump and total water weight

Figure 8 compares the unit water set for each concrete mix with that calculated based on the considerations of the moisture content of LRM. As the LRM content increases, the total water weight increases in the concrete mixtures. In addition, the LRM substitution method has a higher proportion of water in the unit volume than the LRM addition method because some of the reduced cement was filled with water. Therefore, the difference in the concrete slump according to the LRM mixing method and LRM content can be attributed to the influence of the total water weight. The relationship between the total water weight and LRM concrete slump can be clearly identified in Figure 9.

**Figure 10:**
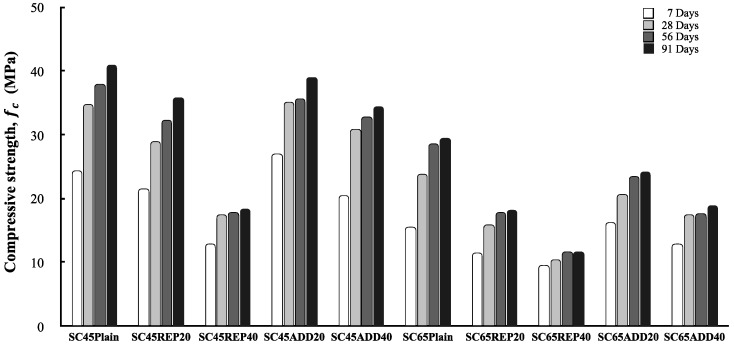
Compressive strength according to the LRM mixing method.

**Figure 11:**
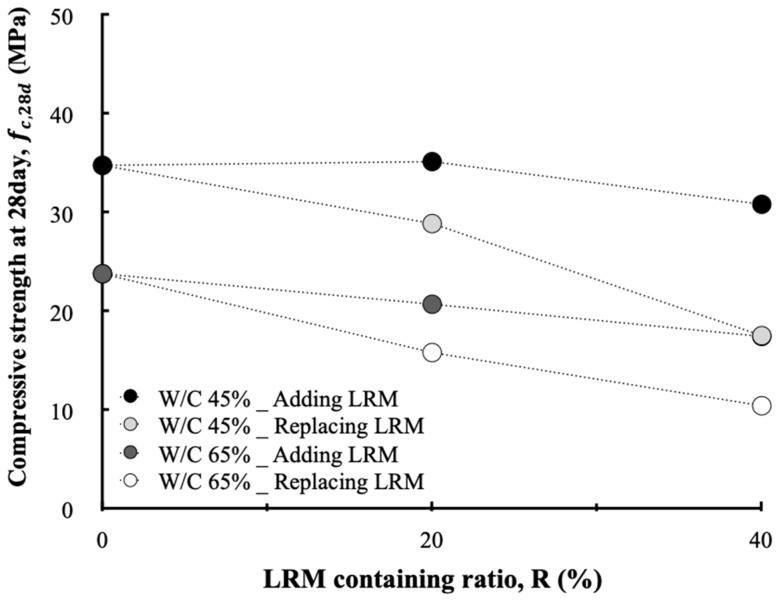
Compressive strength at 28 d according to the LRM containing ratio.

Figure 10 shows the compressive strength of concrete according to the LRM mixing method. The LRM concrete exhibits a general tendency such that the compressive strength increases as a function of age. It was confirmed that the LRM mixing method and LRM content affected the compressive strength of concrete. Figure 11 shows the relationship between the LRM mixing method or the LRM content and the compressive strength of concrete at 28 d of age. First, as the LRM content increased, the compressive strength fc28 of concrete decreased. This result is in agreement with the tendency reported by many previous studies. LRM-substituted concrete yielded a linear reduction in compressive strength as the LRM content increased, but LRM-added concrete exhibited compressive strengths which were similar to those of plain concrete for values up to 20%.

**Figure 12:**
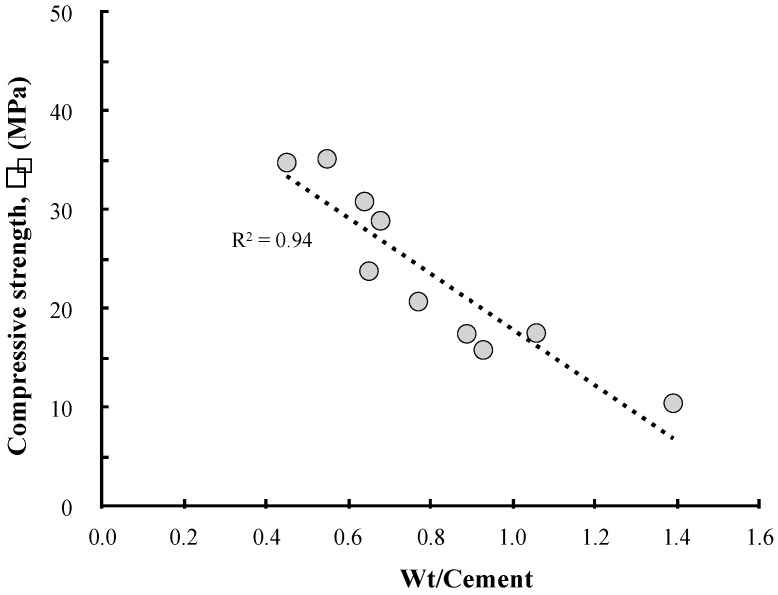
Variation of the compressive strength $f_{c}$ as a function of Wt/cement.

A reduction in the strength of mortar or concrete owing to an increase in the RM content has already been pointed out in many studies. A reduction in the cement content owing to the addition of RM and an increase in the diameters of the capillary pores are considered as the causes that lead to strength changes [15,19]. From this perspective, the LRM substitution method involves an increase in the W/B ratio of concrete because the cement content is reduced, while the water content in the concrete mix is increased. Of course, the LRM addition method has the same problem, but it causes a smaller reduction in compressive strength because the quantities of reduced cement and increased water are relatively smaller compared to the LRM substitution method. This can be confirmed by the relationship between the W/B ratio and the compressive strength of concrete shown in Figure 12. 

Based on these results, it was confirmed that the use of LRM as a concrete admixture caused changes in the unit water and cement content of the concrete mix. In addition, the method of adding LRM is recommended considering the slump and compressive strength of concrete.

### 3.2. Mechanical Properties According to Cement Type

#### 3.2.1. Stress–Strain Curve

**Figure 13:**
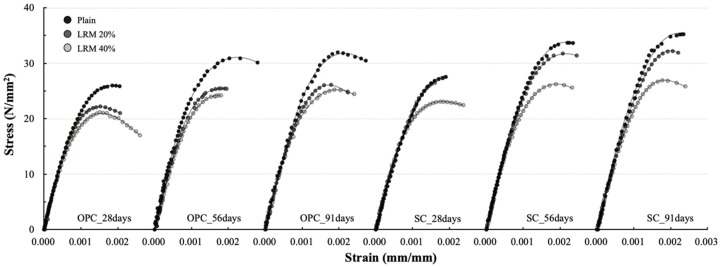
SSCs of LRM concrete for different cement types.

Figure 13 shows the SSCs of concrete according to the cement type and LRM content. All of the SSCs exhibited a typical shape with an upward convex curvature, but the maximum stress and strain were different depending on the cement type and LRM content.

In all of the SSCs, the slope was very steep (up to 70%) of the maximum stress, and the curves were almost linear. It is generally known that the cracks generated from the adhesion relationship between the cement paste and aggregate interface affected the curvature when stresses were less than 70% of the maximum stress [22]. The shapes of the SSCs indicate that the influence of the use of LRM on the cracking behavior of the aggregate interface is not significant. As a matter of fact, it has already been confirmed by the analysis of Tang et al. [17] that the mixing of RM does not cause a significant difference in the properties of the interfacial transition zone (ITZ) between the cement paste and the aggregate.

Meanwhile, the curvature in the straight shape of the SSC occurred earlier as the LRM content increased. The onset of the curvature in the SSC is related to the cracks of mortar and their propagation. Previous studies have reported that the mixing of RM lowers the compressive strength of mortar. Therefore, different curvature starting points depend on the LRM application conditions, and are estimated to be affected by the strength development of mortar owing to the use of LRM.

The ultimate stress of the concrete that used OPC showed a decreasing tendency with the addition of LRM, and the difference due to the LRM content was not large. However, in the case of the concrete that used SC, the ultimate stress did not significantly decrease for LRM contents up to 20%. After the maximum stress, the OPC concrete exhibited a short stress-reduction section, but the SC concrete exhibited brittle behavior as it ruptured when the maximum stress was developed or yielded a shorter stress-reduction section.

#### 3.2.2. Compressive Strength

**Figure 14:**
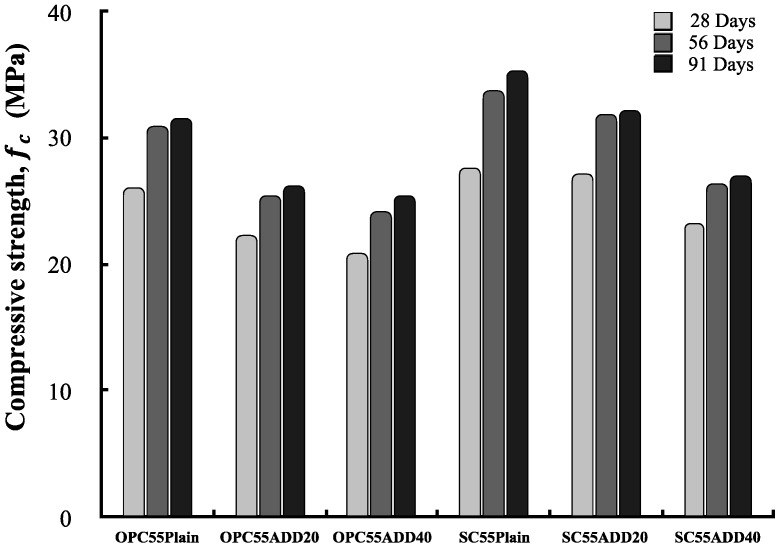
Compressive strength of LRM concrete as a function of cement type.

Figure 14 shows the compressive strength results of LRM concrete according to the cement type. Regardless of the cement type, the compressive strength of concrete showed a tendency to decrease as the LRM content increased, and this tendency was also maintained when the age increased. The compressive strength of SC55ADD20 at 28 d of age was similar to that of SC55plain. SC55ADD20 exhibited the smallest difference in compressive strength after 56 d of age compared with SC55 plain. 

**Figure 15:**
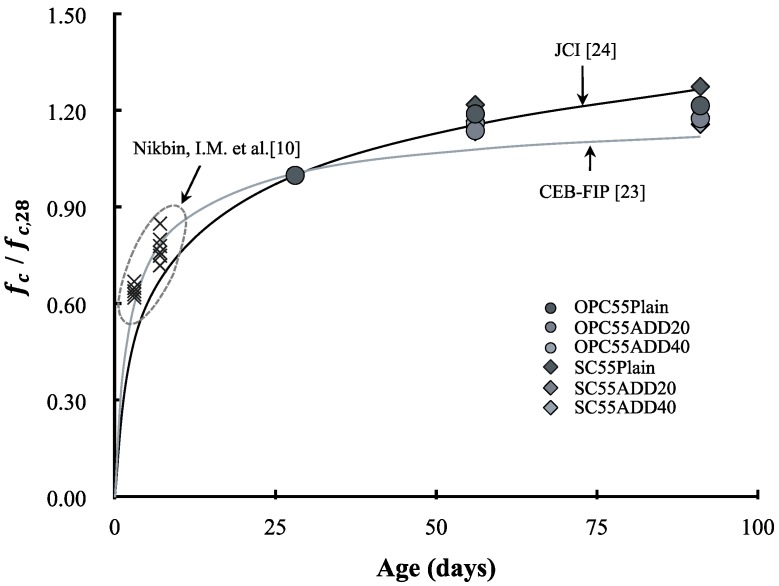
Variation of compressive strength as a function of age.

Figure 15 shows the compressive strength development of LRM concrete, and compares various models (e.g., CEB-FIP [23], JCI [24]). According to Nikbin et al. [10], the compressive strength of the RM concrete develops in a similar manner to the CEP-FIP model up to 28 d of age. In this study, the compressive strengths at 28, 56, and 91 d of age were examined. It was found that the compressive strength of plain OPC developed similarly to the prediction equation of CEB-FIP, and that the long-term compressive strength development of plain SC was higher than that of plain OPC and close to the JCI model. The fact that the long-term compressive strength of SC concrete is higher than that of OPC owing to the latent hydraulic properties of slag powder has already been reported. The compressive strength development of LRM concrete was close to the JCI model at 56 d of age, but it had values which were between the JCI and CEB-FIP models at 91 d.

**Figure 16:**
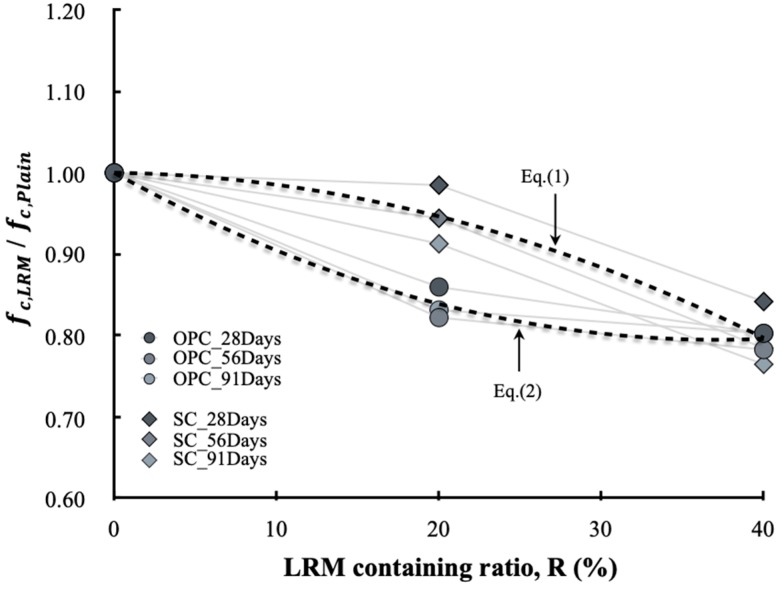
Effect of LRM containing ratio on compressive strength.

Figure 16 shows the effects of the cement type and LRM content on the compressive strength of concrete. The compressive strength of concrete according to the LRM content clearly showed different tendencies depending on the cement type. In addition, the difference was obvious when the LRM 20% was added. The compressive strength of SC55ADD20 was similar to that of SC55Plain at 28 d of age, and was approximately 90% of that of SC55Plain at 91 d. The compressive strength of OPC55ADD20 was approximately 15–20% lower than that of OPC55Plain. In the cases of SC55ADD40 and OPC55ADD40, the difference in the compressive strength reduction rate depended was not significant on the cement type. The reduction in the compressive strength owing to the addition of RM is in agreement with the reported findings in previous studies. The cause is attributed to the reduction in the cement content and the increase in unit water owing to an increase in the LRM content. 

Meanwhile, the difference in the compressive strength of LRM-added concrete depended on the cement type and appeared to be affected by the increased alkali environment generated by the addition of RM. As the RM generally contains approximately 10% Na_2_O, concrete containing RM includes high-alkali components. According to the previous literature [25], alkali metal ions are easily liquefied when high-alkali cement is mixed with water. Therefore, the cement hydration rate and the shapes of hydrates change, while the strength and other engineering properties of concrete are also affected. In particular, hydration products in a high-alkaline environment tend to become like gelatin rather than crystallize. Accordingly, the C-S-H structure is relatively thick and heterogeneous and reduces the long-term concrete strength. This phenomenon is more profound in OPC and in SC.

Therefore, to address the compressive strength reduction problem with concrete that uses RM, it is necessary to determine the appropriate cement type and RM substitution rate. In the range examined in this study, it was confirmed that more than 90% of the compressive strength of the plain type could be developed up to an age of 91 d if SC is used and if 20% LRM is added. In addition, based on the experimental results, the compressive strength development rate according to the LRM content can be expressed with Equations (1) and (2), depending on the cement type.

(1) $$\frac{f_{c,sc+LRM}}{fc,sc}=0.0002R^{2}-0.0111R+1\;$$

(2) $$\frac{f_{c,OPC+LRM}}{f_{c,OPC}}=-0.0001R^{2}-0.0002R+1$$

#### 3.2.3. Elastic Modulus and Peak Stress

**Figure 17:**
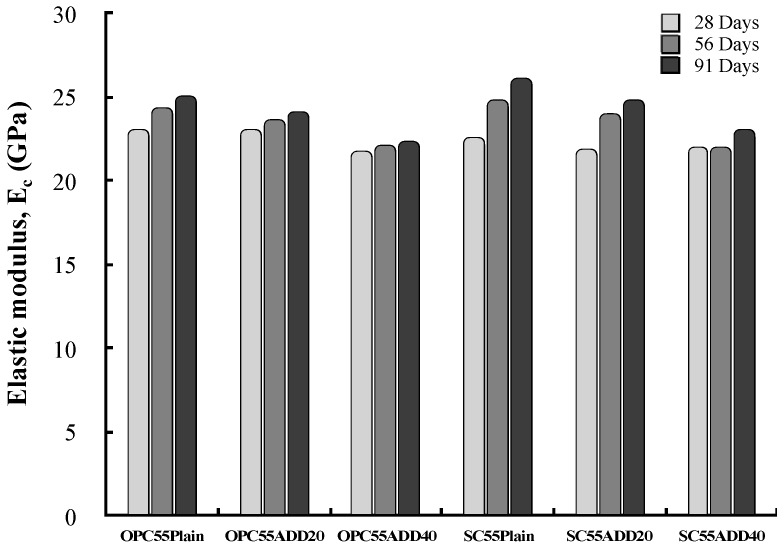
Variation of the elastic modulus of LRM concrete as a function of cement type.

Figure 17 shows the elastic modulus of the LRM-added concrete. Overall, the elastic modulus shows a similar tendency to that of the compressive strength. The elastic modulus was defined as the slope of the straight line which connected the initial point and the stress point (which corresponded to 40% of the maximum stress) in the stress–strain curve. A difference in the elastic modulus resulted according to the LRM addition condition. This difference was not as large as that obtained in the case of compressive strength because the slopes of all specimens (up to 70% of the maximum stress) were not significantly different.

**Figure 18:**
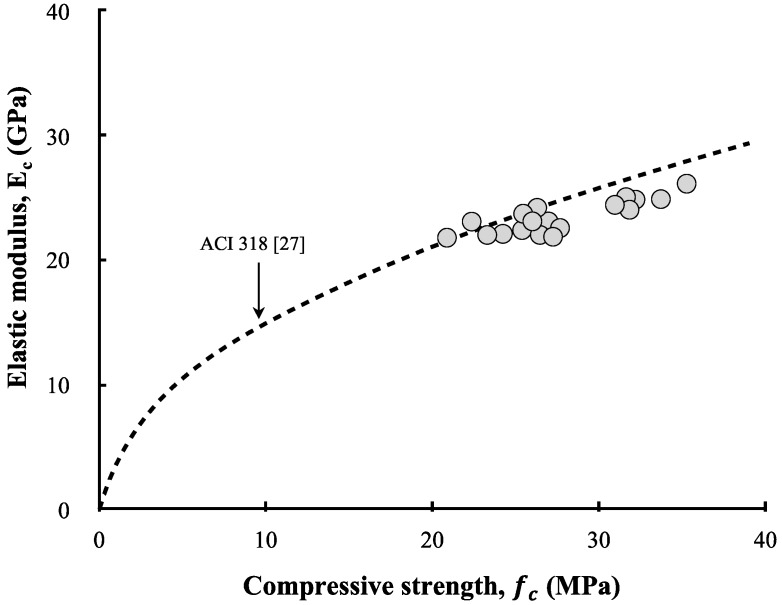
Relationship between elastic modulus and compressive strength.

Figure 18 compares the experimental results based on the ACI 318 [26] model. The elastic moduli of all specimens measured in this study were close to the ACI 318 model. In other words, the elastic modulus of the LRM concrete was found to be proportional to the compressive strength. No special differences were noted owing to the cement type. Klieger [27] and Fulton [28] also reported that the elastic modulus of OPC concrete was not significantly different from that of SC concrete. As the type and quantity of aggregate have dominant impacts on the elastic modulus of concrete, it appears that the elastic modulus was affected by the reduction of the cement and aggregate owing to the LRM addition rather than the cement type.

**Figure 19:**
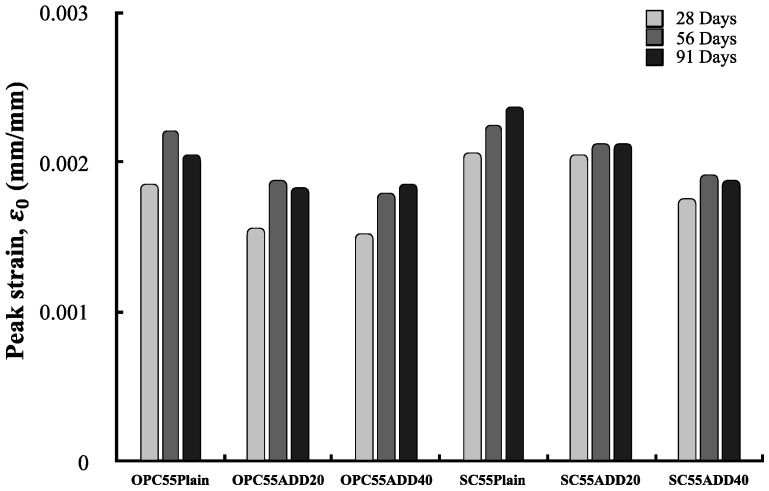
Peak strain of LRM concrete with cement type.

Figure 19 shows the peak strain of LRM-added concrete. The measured peak strain of LRM-added concrete ranged from 0.0015 to 0.0025. This range was the same as the peak strain range of ordinary strength concrete. SC concrete exhibited a higher peak strain compared to OPC concrete, and the peak strain showed a decreasing tendency as the LRM content increased regardless of the cement type. The peak strain has a close relationship with the compressive strength and elastic modulus because it is affected by the strength of the cement matrix and the cracks that occur in the aggregate ITZ. Therefore, it appears that the reduction in the compressive strength of concrete due to the addition of LRM leads to a reduction in peak strain.

**Figure 20:**
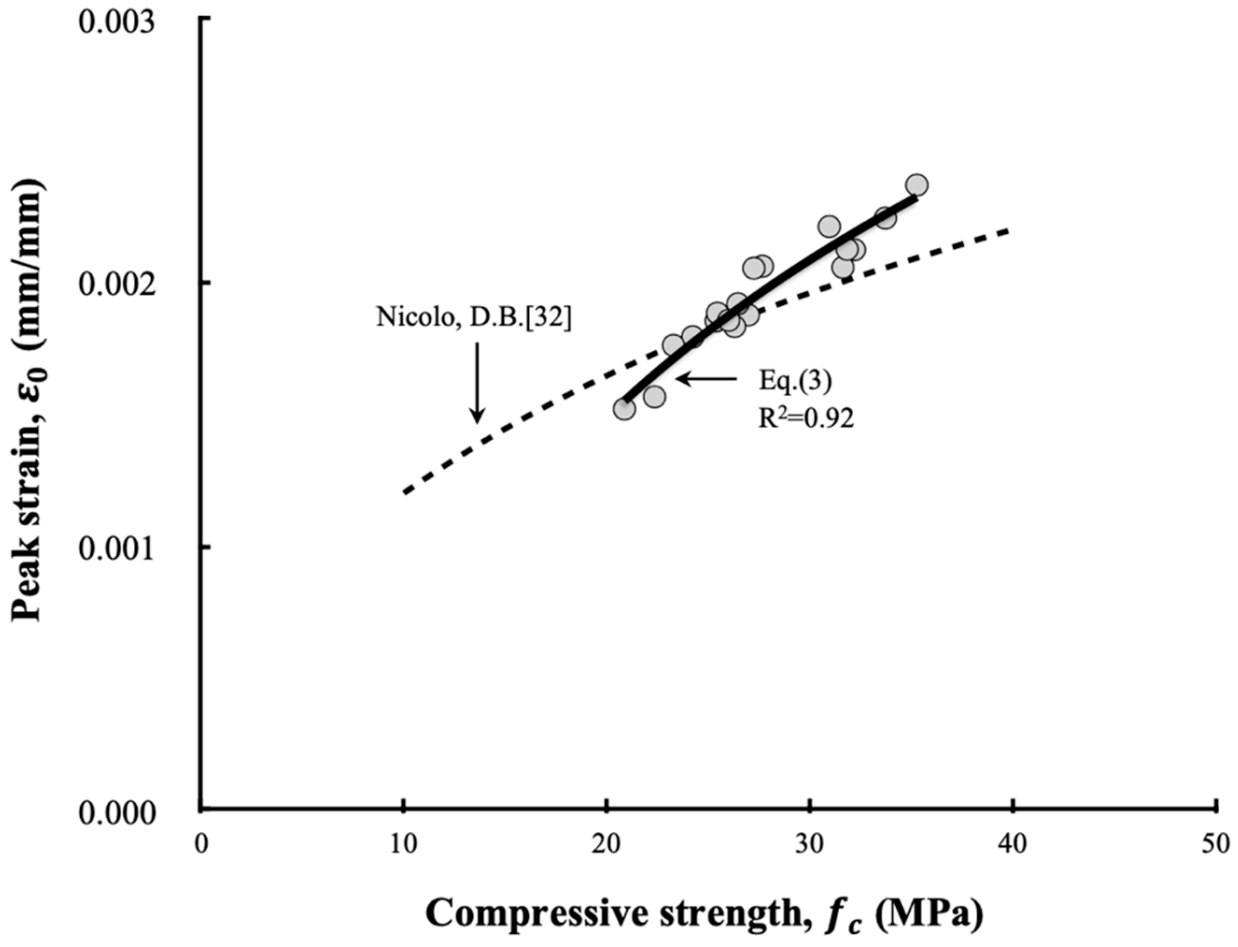
Relationship between peak strain and compressive strength.

Some codes have proposed the value of 0.002 as the peak strain of concrete in the case of uniaxial loading. However, the peak strain actually varies depending on the concrete mix, curing condition, shape and size of the specimen, loading rate, age, and experimental equipment [29,30]. Nevertheless, Tasdemir et al. [31] confirmed that there is a specific relationship between the compressive strength and peak strain based on the analyses of various experimental data, while Nicolo [32] proposed a relationship between the compressive strength and peak strain of ordinary concrete. The relationship between the compressive strength and peak strain could also be confirmed in this study based on the measured results. It is difficult, however, to assess whether the relationship is consistent with that proposed by Nocolo’s equation, as shown Figure 20. Therefore, based on the experimental results, the relationship between the compressive strength and peak strain of LRM-added concrete can be formulated according to Equation (3).

(3) $$\varepsilon_{0}=0.0015\ln\left(f_{c}\right)-0.0029,$$

where $\varepsilon_{o}$ is peak strain and $f_{c}$ is the concrete’s compressive strength (MPa).

## 4. Approximation of the Stress–Strain Relations

**Figure 21:**
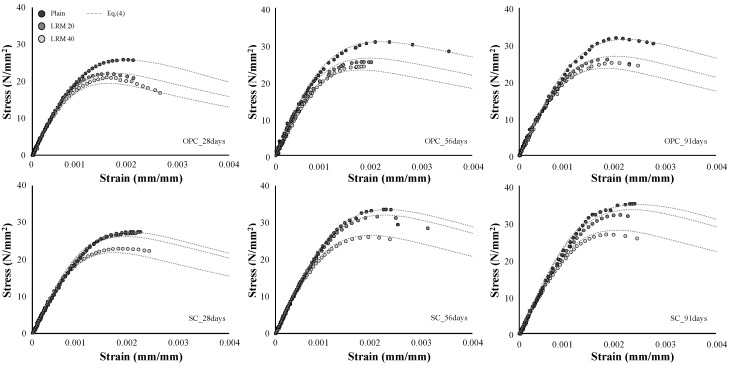
Approximation of the stress–strain relation curve in the case of LRM concrete.

Figure 21 compares the experimental results with the SSCs calculated based on the use of a model. Equation (4) from Carreira and Chu [33] was used as the basic model for approximating the SSCs. In this equation, *β* that determines the shape of the SSC exhibits no special tendency according to the cement type and LRM addition condition. The value of *β* was fixed to two. The variable fc was calculated with Equations (1) and (2), and the peak strain was estimated from the calculated fc using Equation (3). Based on the equation estimated by the LRM content, the approximate SSCs which were relatively similar to the experimentally obtained SSCs could be derived. Therefore, the approximate stress–strain relationship of LRM concrete can be used as a reference for various engineering areas.

(4) $$\frac{\sigma_{c}}{f_{c}}=\frac{\beta \frac{\varepsilon_{c}}{\varepsilon_{0}}}{\beta -1+{\left(\frac{\varepsilon_{c}}{\varepsilon_{0}}\right)}^{\beta }},$$

where $\sigma_{c}$ is the stress, $f_{c}$ is the ultimate stress, $\varepsilon_{c}$ is the strain, and $\varepsilon_{0}$ is the peak strain.

In this study, however, the descending phase could not be estimated because data could not be collected. In the SSC of the descending concrete, the descending phase after the ultimate stress is affected by the experimental equipment and is not recognized as a material property, but the nonlinear structural analysis of concrete structures, such as the seismic design, may require the descending phase section. Therefore, in future research, more detailed verification will be necessary based on the comparison between the experimental data of this section and the approximation formula.

## 5. Conclusions

1. In this study, LRM was fabricated by mixing RM sludge, water, a thickener, and an antifoamer. When LRM was used as an admixture for concrete, there was no degradation in the fluidity of concrete that used conventional RM because the moisture reduction that affected the fluidity of the concrete mix could be prevented

2. Concrete mixed with LRM exhibited problems associated with the increased W/B ratio and the decreased mechanical properties because the cement content decreased, and the water content increased compared to the designed mix. Therefore, the LRM addition method is recommended compared to the replacement method in which some cement was replaced with LRM based on considerations of changes in the fluidity and mechanical properties of concrete

3. Owing to the influence of the high-alkaline environment generated by the addition of RM on the hydration products of cement, the mechanical properties of the LRM-added concrete varied depending on the cement type. Based on the experiment results, it is recommended that SC uses LRM as the concrete admixture and an LRM content of 20% with respect to the weight of the cement

4. From the relationship between the compressive strength and peak strain according to the cement type and LRM content, the stress–strain relationship of the LRM concrete that can be used in engineering areas could be approximated. As the LRM content examined in this study had a limited range and the stress–strain relationship of concrete was affected by various mixing factors, it is necessary to use the results which had been verified experimentally given that they emulate accurately the required conditions when accurate stress–strain relationships are required

## Figures and Tables

**Table 1:** Physical properties and chemical composition of red mud sludge.

Material	Density (g/cm^3^)	Average Diameter (μm)	Viscosity (cP)	Water Content (%)	Chemical Composition (%)						
					SiO_2_	Al_2_O_3_	Fe_2_O_3_	CaO	MgO	Na_2_O	K_2_O
RM sludge	2.0	4.31	20,000	50.2	38.8	16.1	22.8	3.4	0.2	10.0	0.4

**Table 2:** Physical properties of thickener and antifoamer.

Materials	Type	Color	Viscosity (cPs)	Water Content (%)	pH	Density (g/cm^3^)
Thickener	Methyl Cellulose	White	32,900	1.4	-	-
Antifoamer	Polyoxyalkylene alkylether	-	-	-	4.5~7.5	0.9884

**Table 3:** Mix proportion used for the manufacturing of liquefied red mud.

Material	RM sludge	Water	Thickener	Antifoamer
Proportion (by weight for RM sludge)	1	0.5	0.0036	0.0014

**Table 4:** Physical properties and chemical composition of cement.

Type of Cement	Specific Surface Area (cm^2^/g)	Density (g/cm^3^)	Lg. Loss (%)	Chemical Composition (%)					
				SiO_2_	Al_2_O_3_	Fe_2_O_3_	CaO	MgO	SO_3_
OPC	3140	3.15	1.01	21.3	5.4	3.5	62.0	3.3	1.64
SC	3959	3.06	1.49	27.2	9.4	2.2	52.0	3.6	2.10

**Table 5:** Aggregate properties.

Type of Aggregate	Type	Maximum Size (mm)	Density (g/cm^3^)	Absorption (%)
Fine aggregate	Crushed sand	5	2.56	1.01
Coarse aggregate	Granite	20	2.67	1.39

**Table 6:** Experimental plan and concrete mixture proportion.

Specimen	Cement Type	W/B ^(1)^ (%)	LRM Addition ^(2)^		S/A (%)	Water (kg/m^3^)	Unit Weight(kg/m^3^)					LRM (kg/m^3^)
			Method	R(%)			Binder			S	G	
							OPC	SC	LRM			
SC45Plain	SC	45	-	0	50	135	0	300	0	961	995	0
SC45REP20	SC	45	REP.	20	50	135	0	240	60	935	968	0
SC45REP40	SC	45	REP.	40	50	135	0	180	120	908	940	0
SC45ADD20	SC	45	Add.	20	50	135	0	300	0	961	995	60
SC45ADD40	SC	45	Add.	40	50	135	0	300	0	961	995	120
SC65Plain	SC	65	-	0	50	195	0	300	0	884	915	0
SC65REP20	SC	65	REP.	20	50	195	0	240	60	858	888	0
SC65REP40	SC	65	REP.	40	50	195	0	180	120	832	861	0
SC65ADD20	SC	65	ADD.	20	50	195	0	300	0	884	915	60
SC65ADD40	SC	65	ADD.	40	50	195	0	300	0	884	915	120
OPC55Plain	OPC	55	-	0	50	135	300	0	0	934	967	0
OPC55ADD20	OPC	55	ADD.	20	50	135	300	0	0	934	967	60
OPC55ADD40	OPC	55	ADD.	40	50	135	300	0	0	934	967	120
SC55Plain	SC	55	-	0	50	135	0	300	0	923	955	0
SC5ADD20	SC	55	ADD.	20	50	135	0	300	0	923	955	60
SC55ADD40	SC	55	ADD.	40	50	135	0	300	0	923	955	120

**^(1)^** B: Binder, **^(2)^** R: Ratio (% by cement weight ), REP.: Replacement, ADD.: Addition.
